# Asymmetry of the Active Site Loop Conformation between Subunits of Glutamate-1-semialdehyde Aminomutase in Solution

**DOI:** 10.1155/2013/353270

**Published:** 2013-07-31

**Authors:** Barbara Campanini, Stefano Bettati, Martino Luigi di Salvo, Andrea Mozzarelli, Roberto Contestabile

**Affiliations:** ^1^Dipartimento di Farmacia, Università di Parma, Parco Area delle Scienze 23/A, 43124 Parma, Italy; ^2^Dipartimento di Neuroscienze, Università di Parma, Via Volturno 39, 43125 Parma, Italy; ^3^Istituto Nazionale di Biostrutture e Biosistemi, Viale Medaglie d'Oro 305, 00136 Roma, Italy; ^4^Dipartimento di Scienze Biochimiche “A. Rossi-Fanelli”, Sapienza Università di Roma, Piazzale Aldo Moro 5, 00185 Roma, Italy

## Abstract

Glutamate-1-semialdehyde aminomutase (GSAM) is a dimeric, pyridoxal 5′-phosphate (PLP)- dependent enzyme catalysing in plants and some bacteria the isomerization of L-glutamate-1-semialdehyde to 5-aminolevulinate, a common precursor of chlorophyll, haem, coenzyme B_12_, and other tetrapyrrolic compounds. During the catalytic cycle, the coenzyme undergoes conversion from pyridoxamine 5′-phosphate (PMP) to PLP. The entrance of the catalytic site is protected by a loop that is believed to switch from an open to a closed conformation during catalysis. Crystallographic studies indicated that the structure of the mobile loop is related to the form of the cofactor bound to the active site, allowing for asymmetry within the dimer. Since no information on structural and functional asymmetry of the enzyme in solution is available in the literature, we investigated the active site accessibility by determining the cofactor fluorescence quenching of PMP- and PLP-GSAM forms. PLP-GSAM is partially quenched by potassium iodide, suggesting that at least one catalytic site is accessible to the anionic quencher and therefore confirming the asymmetry observed in the crystal structure. Iodide induces release of the cofactor from PMP-GSAM, apparently from only one catalytic site, therefore suggesting an asymmetry also in this form of the enzyme in solution, in contrast with the crystallographic data.

## 1. Introduction

Chlorophyll, haem, coenzyme B_12_, and other tetrapyrrolic compounds share the same biosynthetic origin. They are assembled from four molecules of porphobilinogen, itself the product of the linkage of two 5-aminolevulinate (ALA) molecules [1]. In animals, yeast, and some bacteria, ALA is synthesised in the Shemin pathway, by the condensation of succinyl-CoA with glycine, followed by decarboxylation. The overall reaction is catalysed by ALA synthase, a pyridoxal 5*'*-phosphate-dependent enzyme [2]. Plants, green algae, and the majority of bacteria synthesise ALA in a completely different way. In these organisms, ALA is obtained through the isomerisation of glutamate-1-semialdehyde (GSA), a compound deriving from glutamate [3]. This reaction is catalysed by glutamate-1-semialdehyde aminomutase (GSAM, EC 5.4.3.8), an enzyme belonging to fold type I PLP-dependent enzymes. The primary sequence and tertiary structure of GSAM are strongly related to the class III of aminotransferases [4]. GSAM is a potential target for safe selective herbicides and antibiotics because, while it carries out an essential role in the metabolism of plants and some pathogenic bacteria, it is absent in animals [5].

**Figure 1:**
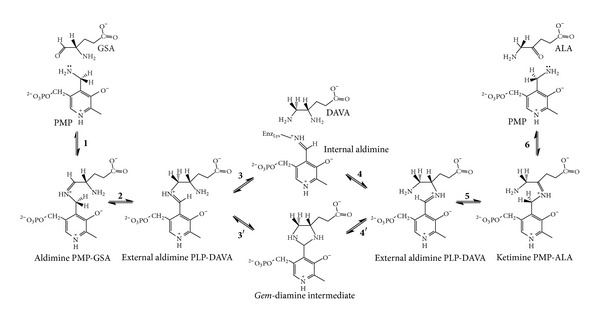
Proposed mechanism for the reaction catalysed by GSAM.

The mechanism of the aminomutase reaction catalysed by GSAM [6–8] is also closely analogous to that of the transamination reaction [9]. The exceptional feature of GSAM is that, unlike aminotransferases, it catalyses an intramolecular exchange of the amino and carbonyl moieties, which are both present in the substrate. The catalytic cycle starts with GSA and the pyridoxamine 5*'*-phosphate (PMP) form of the enzyme and is completed without the need of a cognate amino acid substrate (Figure 1). A pivotal step in the mechanism is the isomerisation between the two aldimine intermediates that can be formed with either the 4 or the 5-amino group of the 4, 5-diaminovalerate (DAVA) intermediate. Whether this part of the reaction takes place through dissociation of DAVA (steps 3 and 4 in Figure 1) or formation of a geminal diamine intermediate (steps 3*'* and 4*'* in Figure 1) is unclear. Dissociation of DAVA appears mechanistically unnecessary, and even detrimental, although some dissociation has been found to occur to an extent governed by the concentration of the enzyme itself [10]. The PLP form of the enzyme is unable to catalyse the aminomutase reaction [10].

At the end of the purification procedure, dimeric GSAM from *Synechococcus* contains a mixture of PLP and PMP [7, 11]. This is invariably observed in GSAM purified from other sources [8, 12]. Preparations of native GSAM can be treated with substrate analogues that have either an amino or a carbonyl function and, therefore, react in a half-transamination reaction, converting the enzyme into either the PLP form or the PMP form [13]. Notably, crystallographic investigations on native GSAM from *Synechococcus* showed that the distribution of PLP and PMP was asymmetric within each enzyme dimer [14, 15]. Moreover, the two subunits showed asymmetry also in the mobility of a 19-residue loop (spanning residues 153–181), which has been shown to control access to the active site and limit the dissociation of the DAVA intermediate [16]. Crystallographic studies have been also carried out on the apoenzyme form and on double PLP or double PMP forms (containing the same form of cofactor in both subunits of the same dimer and obtained by adding either PLP or PMP to the apoenzyme) [15]. In the apo form, the active site loop is disordered in both subunits, whereas in the double PMP form, it is ordered and symmetrically opened. In the double PLP form, one loop is closed and the other is disordered (Table 1). It seems that the loop cannot be present in a closed conformation in both subunits at the same time. It is clear that the conformational state of the loop depends on the particular form of the cofactor present at the active site and is functional to the reaction trajectory of the aminomutase reaction catalysed by GSAM [15, 17]. When PMP is present at the active site, the loop is open, thus allowing substrate entry and product release. In contrast, when the active site contains PLP as internal aldimine and the intermediate DAVA, the loop is closed and access to the active site is obstructed by the short helical section of the loop residues 164–168 [15].

Since no studies on the structural and functional asymmetry of GSAM in solution have been reported in the literature, we investigated the effect of different fluorescence quenchers on the emission properties of the *Synechococcus *enzyme in solution, in order to ascertain the existence of a relationship between the form of the cofactor present at the active site and its accessibility.

## 2. Materials and Methods

### 2.1. Materials

GSA aminomutase from *Synechococcus* was expressed and purified as described previously [11]. All chemicals and buffers were purchased from Sigma-Aldrich (St. Louis, MO, USA) and were of the best available quality.

### 2.2. Conversion of the Native Enzyme into the PLP and PMP Form

Complete conversion of the enzyme into the PLP or PMP form was achieved using succinic semialdehyde and racemic 4, 5-diaminovalerate (DAVA), respectively, and followed by gel filtration. Succinic semialdehyde (20 mM) or DAVA (5 mM) was added to a solution (1 mL) of enzyme (60–300 *μ*M) in 100 mM Na-Tricine buffer, pH 7.9. Succinic semialdehyde or DAVA in the same buffer (1 mL, same concentrations) was loaded on a G-25 Sephadex column (45 × 1 cm) and allowed to drain in before loading the enzyme. The column was equilibrated and eluted with 100 mM Na-Tricine, pH 7.9. The high molecular mass fraction was collected in the void volume well separated from small molecules.

### 2.3. Absorbance and Fluorescence Spectroscopy

PLP-GSAM and PMP-GSAM, stored as concentrated solutions in 100 mM Tricine pH 7.9, were diluted in 20 mM Tricine pH 7.9 for fluorescence and absorption spectroscopy experiments. Experiments were carried out at 25°C.

Absorption measurements were carried out using a Cary Scan 400 spectrophotometer (Varian Inc., Palo Alto, CA, USA). Temperature was maintained at 25 ± 0.5°C with a circulating water bath.

The fluorescence emission of tryptophans and the four cofactor species under analysis were detected by exciting at 298 nm, 320 nm, 330 nm, 340 nm, and 420 nm, respectively, using a FluoroMax-3 fluorometer (HORIBA Jobin Yvon, Longjumeau Cedex, France) equipped with a thermostated cell holder. Excitation and emission slits were set at 3 nm except for excitation at 420 nm where 5 nm slits were used. Spectra were corrected for buffer contribution. The accessibility of the cofactor was assessed by fluorescence quenching. CsCl, KI, and acrylamide solutions were prepared in 20 mM Tricine, pH 7.9. Na_2_S_2_O_3_ at 0.01 mM concentration was added to KI solutions to prevent iodide oxidation. Fluorescence of either 6 *μ*M PLP-GSAM or 2 *μ*M PMP-GSAM was excited at 330 nm using 5 nm slits. Quenching data were analyzed with the Stern-Volmer equation [18]:

(1) $$\begin{array}{c} \frac{F_{0}}{F}=1+K_{\text{SV}}\cdot \left[Q\right], \end{array}$$

where *F*_0_ is the fluorescence intensity in the absence of the quencher, *F* is the fluorescence at each given quencher concentration, *K*_ SV_ is the Stern-Volmer constant, and [*Q*] is the concentration of the quencher.

### 2.4. Time-Resolved Fluorescence Spectroscopy

Fluorescence intensity decays were measured by the phase and modulation technique [19, 20] using an ISS GREG 200 fluorometer (ISS Inc., Champaign, IL, USA). PLP-GSAM fluorescence decays were measured at a protomer concentration of 48.8 *μ*M, upon excitation at 330 nm performed using a 300 W xenon lamp and a Jobin Yvon monochromator with 16 nm band width. A *p*-bis [2-(phenoxazoyl)] benzene (POPOP) solution was used as a lifetime standard reference (*τ* = 1.05 ns). To eliminate polarization artifacts in the intensity decay, data were collected under magic angle conditions with the excitation light polarized normal to the laboratory plane, 0°, and the emission polarizer oriented at 54.7° [20]. Samples were equilibrated at 25 ± 0.5°C using a jacketed cell holder and a circulating water bath. A set of 20 modulation frequencies, varied continuously in the 2 to 200 MHz range, was used. Data were fitted to a sum of discrete exponentials [21] with lifetime *τ*_*i*_ and fractional intensity *f*_*i*_ by the Marquardt algorithm of the Globals Unlimited software (University of Illinois, Urbana, IL, USA) [22]. Frequency-independent standard error values of 0.2° for phase data and 0.004 for modulation data were routinely applied. The *χ* ^2^ minimization was the criterion used to select the best fits [19, 21]. The *τ*_*i*_ and *f*_*i*_ values reported in Results and Discussion represent the average of three measurements on different samples.

Mean lifetimes 〈*τ*〉 were obtained from lifetimes *τ*_*i*_ and fractional intensities *f*_*i*_ according to the equation [23]:

(2) $$\begin{array}{c} \langle \tau \rangle =\sum \alpha_{i}\cdot \tau_{i}, \end{array}$$

where *α*_*i*_, the preexponential factor for a multiexponential decay law, was determined as

(3) $$\begin{array}{c} \alpha_{i}=\frac{f_{i}/\tau_{i}}{\sum f_{i}/\tau_{i}}. \end{array}$$

## 3. Results and Discussion

### 3.1. Spectroscopic Properties of PLP-GSAM and PMP-GSAM

**Figure 2:**
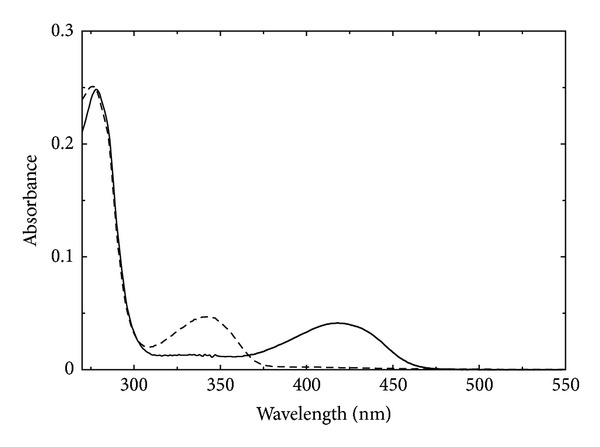
Absorption spectra of PLP-GSAM and PMP-GSAM. Absorption spectra of PLP-GSAM (*solid line*) and PMP-GSAM (*dashed line*) recorded on a solution containing 7.1 *μ*M protein in 20 mM Tricine, pH 7.9.

Two freshly prepared forms of the enzyme from *Synechococcus* were used: the PLP form (PLP-GSAM), obtained by treating the native enzyme with succinic-1-semialdehyde, and the PMP form (PMP-GSAM), obtained by the addition of DAVA [13]. The absorption spectra of equimolar solutions of PLP-GSAM and PMP-GSAM are shown in Figure 2. The absorption spectrum of PLP-GSAM shows, in addition to the band of aromatic amino acids centred at 278 nm, a major band centred at 418 nm, typical of the internal aldimine between PLP and the active site lysine, and a modest though measurable absorbance at about 330 nm that could be attributed to the enolimine tautomer of the internal aldimine or to traces of PMP bound to the active site of the enzyme. Several PLP-binding enzymes exhibit equilibrium between enolimine and ketoenamine tautomers of the Schiff base formed by the coenzyme with the active site lysine (internal aldimine) or with a bound amino acid or reaction intermediate (external aldimines). Ketoenamine tautomers absorb light at longer wavelength with respect to enolimine and are favored in more polar active sites [24]. PMP-GSAM shows a band at 340 nm attributed to PMP in the active site of the enzyme [6]. No absorption from the internal aldimine of PLP is discernible in the visible range of the spectrum. The absorption spectrum confirms that PMP-GSAM is pure with respect to the content of the cofactor.

**Figure 3:**
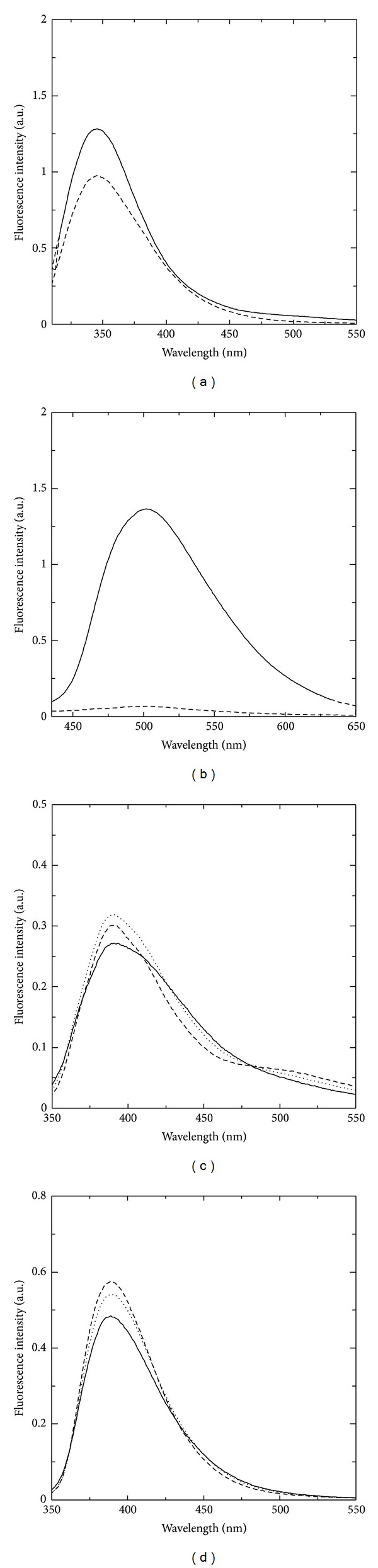
Fluorescence emission spectra of PLP-GSAM and PMP-GSAM. To allow direct comparison of the data, the reported spectra are normalized for protein concentration. Spectra were collected in 20 mM Tricine buffer, pH 7.9 solutions and were normalized to a protein concentration of 7 *μ*M. (a) Emission spectra of PLP-GSAM (*solid line*) and PMP-GSAM (*dashed line*) upon excitation at 298 nm with 3 nm emission and excitation slits. (b) Emission spectra of PLP-GSAM (solid line) and PMP-GSAM (dashed line) upon excitation at 420 nm with 5 nm emission and excitation slits. (c) Emission spectra of PLP-GSAM upon excitation at 320 nm (solid line), 330 nm (dotted line), and 340 nm (dashed line) with 3 nm emission and excitation slits. (d) Emission spectra of PMP-GSAM upon excitation at 320 nm (solid line), 330 nm (dotted line), and 340 nm (dashed line) with 3 nm emission and excitation slits.

The intrinsic fluorescence of tryptophans and coenzyme has been previously exploited to investigate structural and dynamic features of some PLP-dependent enzymes and their modification upon binding of substrates, inhibitors, and regulatory molecules [25–30]. In GSAM, both PLP and PMP are fluorescent upon excitation in either the UV or visible range of the spectrum. At 298 nm, tryptophan fluorescence is selectively excited, with almost no contribution from the other aromatic side chains of tyrosine and phenylalanine. When excited at 298 nm, both PLP-GSAM and PMP-GSAM show an emission band centred at about 345 nm characteristic of direct tryptophan emission (Figure 3(a)). Emission from PMP-GSAM is slightly less intense than that of PLP-GSAM. GSAM has two tryptophans, Trp53 and Trp67. Trp67 is buried inside the protein structure at about 10 Å from the cofactor, whereas Trp53 is exposed to the solvent far away from the active site. The distance between Trp67 and the coenzyme is compatible with Förster resonance energy transfer from the former to the latter [25, 31]. However, differently from what observed for other PLP-dependent enzymes like *O*-acetylserine sulfhydrylase [25] or tryptophan synthase [32], the perpendicular spatial disposition of the two chromophores appears to hinder an efficient energy transfer, as demonstrated by the emission spectrum upon excitation at 298 nm where no bands around 500, attributable to coenzyme emission, are observed, except for a barely detectable shoulder in the case PLP-GSAM (Figure 3(a)).

Upon excitation at 420 nm, only PLP-GSAM yields a significant emission, with a band centered at 500 nm (Figure 3(b)). In agreement with absorption data, this observation further supports the notion that PMP-GSAM samples do not carry significant amounts of cofactor in the PLP form.

Both PMP-GSAM and PLP-GSAM show fluorescence emission upon excitation at around 330 nm (Figures 3(c) and 3(d)). The shape of PMP-GSAM emission spectrum does not depend on the excitation wavelength in the 320–340 nm range, and the emission intensity is maximum at *λ*_ex_ = 340 nm, consistent with the absorbance spectrum (Figure 2). The emission spectra of PLP-GSAM for excitation at 320, 330, and 340 nm also show a broad band centred at about 390 nm, that could be due to the direct emission of the enolimine tautomer of PLP or to the presence of some fraction of subunits carrying a chromophore in the PMP form. If the latter was predominantly the case, we would expect fluorescence emission to parallel the behaviour of PMP-GSAM, with emission intensity increasing consistently from *λ*_ex_ = 320 nm to *λ*_ex_ = 340 nm. Moreover, the emission band of PLP-GSAM excited at 320 and 330 nm appears to have a slightly red-shifted (by 3-4 nm) peak wavelength with respect to PMP-GSAM, and is definitely more asymmetric, being broader on the low energy side of the emission spectrum (the ratio of emission intensity at 390 and 500 nm is about 5, compared to more than 20 for PMP-GSAM). Furthermore, differently from PMP-GSAM, when PLP-GSAM is excited at 340 nm, the main emission band is slightly decreased in intensity and is accompanied by a shoulder at about 500 nm (Figure 3(c)), most likely due to direct excitation of the high energy tail of the absorption band of PLP ketoenamine tautomer [30, 33, 34]. The presence of a discrete emission at around 500 nm could also originate from the ketoenamine tautomer that forms in the excited state following proton transfer from the 3*'*-OH group to the imine nitrogen [35, 36]. This observation further supports the view that the absorption at around 330 nm arises from the enolimine tautomer of the internal aldimine rather than from a substituted aldimine, as previously pointed out for other PLP-dependent enzymes [35, 37].

The results of absorbance and fluorescence emission spectroscopic characterization clearly indicate that the PLP-GSAM and PMP-GSAM preparations used in this work are at least 90% pure with respect to the state of the bound cofactor.

### 3.2. Fluorescence Quenching

**Figure 4:**
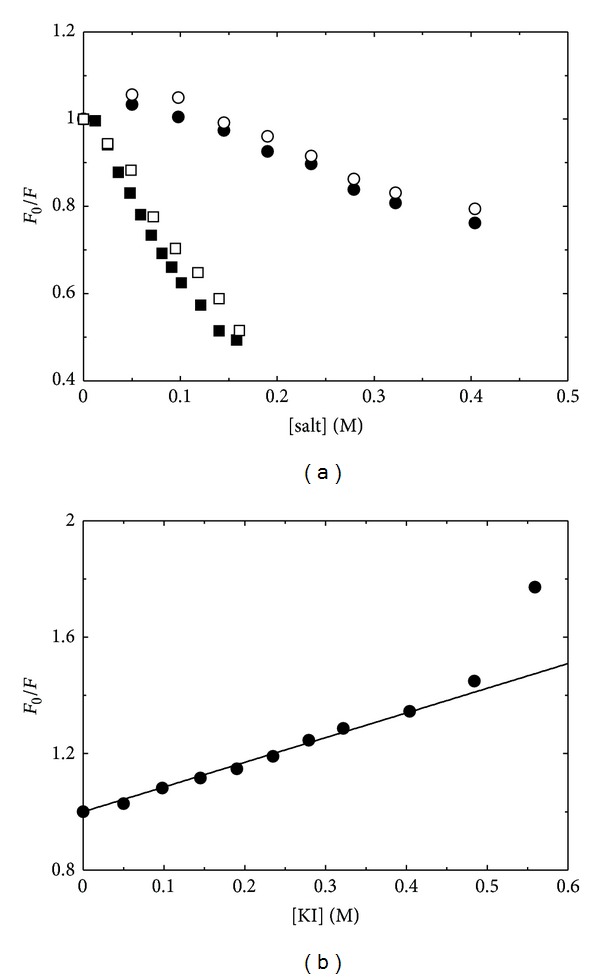
Dependence of fluorescence emission intensity on quencher concentration. (a) Dependence of the relative emission intensity at 384 nm, upon excitation at 330 nm, of PLP-GSAM (circles) and PMP-GSAM (squares) on caesium chloride (closed symbols) and potassium chloride (open symbols) concentration. (b) Stern-Volmer plot for the quenching of PLP-GSAM by iodide. The line through data points represents the fitting to (1).

Fluorescence quenching is a powerful technique to assess the accessibility of fluorophores to the solvent. We have previously exploited this approach to investigate changes in cofactor accessibility of wild-type and mutant forms of another PLP-dependent enzyme, *O*-acetylserine sulfhydrylase [38]. Quenchers that are commonly used in protein fluorescence are caesium (as chloride salt), iodide (as potassium salt), and acrylamide. The different polarity and charge of these molecules allow the application of fluorescence quenching to assess the accessibility of both apolar and polar sites. In fact, the polarity of the site entrance influences the access of the quencher independently of the steric accessibility of the site. The active site of GSAM is mainly edged by positively charged residues likely to hamper the access to the positively charged caesium ions [14]. Quenching experiments were carried out exciting the fluorescence of the cofactor at 330 nm. Both acrylamide and caesium chloride proved to be ineffective at quenching the cofactor fluorescence. In particular, whereas acrylamide does not show any effect on the emission intensity of the cofactor up to about 1 M, caesium chloride induces an increase, rather than a decrease, in the cofactor emission intensity of both PMP-GSAM and PLP-GSAM (Figure 4(a)). The same effect is observed if caesium chloride is substituted by potassium chloride (Figure 4(a)) or potassium acetate (data not shown), suggesting a nonspecific effect of the ionic strength on the fluorescence quantum yield of the cofactor. The effect is more pronounced on PMP-GSAM, leading to a doubling of the signal at about 140 mM salt. 

On the other hand, potassium iodide is effective in quenching the fluorescence of PLP-GSAM (Figure 4(b)), indicating that at least one catalytic site is in an open conformation, accessible to the anionic quencher. The linearity of data shown in Figure 4(b) demonstrates that the enolimine form of enzyme-bound PLP is exposed to a structurally homogeneous environment. The ionic radius of Cs^+^ is about 1.6 Å whereas the ionic radius of I^−^ is about 2.2 Å, so the differences in the accessibility to the active site cannot be ascribed to steric effects but rather to electrostatic repulsion between Cs^+^ and polar or positively charged groups at the active site entrance. The analysis of experimental data in Figure 4(b) according to (1) (Materials and Methods) gave a Stern-Volmer constant (*K*_SV _) value of 0.84 ± 0.01 M^−1^. Since the value of *K*_SV_ that we measured for quenching by iodide of free PLP in solution is 2.61 ± 0.07 M^−1^, in agreement with data present in the literature [39], it appears that the active site of PLP-GSAM is only partially accessible to the solvent. More significantly, the difference between the bimolecular quenching rate constants (i.e., the ratio between *K*_SV_ and the lifetime of the excited state [39–41]) of free PLP and PLP-GSAM complexes, that are the true measure of active site accessibility, should be larger than the difference between the Stern-Volmer constants, considering that the fluorescence lifetime of free PLP is expected to be significantly smaller than that of protein-bound PLP. The fluorescence lifetime decays of PLP-GSAM are well fitted by two discrete exponential components with lifetimes of 8.6 ± 0.2 and 1.40 ± 0.05 ns and fractional intensity of 10.3 ± 0.8% and 89.7 ± 0.8%, respectively, (data not shown). The mean lifetime, calculated as reported in Materials and Methods, is 1.53 ns. This value can be compared to those calculated from data reported in the literature [25] for free PLP in solution and PLP-L-valine, a model compound of PLP Schiff base, upon excitation at 330 nm : 0.32 ns and 0.68 ns, respectively. By considering the latter a better mimic of PLP bound to the enzyme as an internal aldimine, a ratio of about 7 can be calculated between the apparent bimolecular quenching rate constant of PLP-L-valine (3.84 · 10^−9^ M^−1^ s^−1^) and PLP-GSAM (0.55 · 10^−9^ M^−1^ s^−1^). These values are in good agreement with those previously reported for free coenzyme and the internal aldimine of the PLP-dependent enzyme tryptophan synthase *α*_2_ *β*_2_ complex (0.52 · 10^−9^ M^−1^ s^−1^) [39], known to be in an “open” conformation [42].

At potassium iodide concentrations higher than 0.5 M, the Stern-Volmer plot of PLP-GSAM quenching by iodide shows an upward curvature (Figure 4(b)) that could be due to a static quenching component or to a conformational change in the protein structure that leads to a change in the accessibility of the active site to the quencher. For example, an upward curvature of the Stern-Volmer plot of the tetrameric PLP-dependent enzyme tryptophan synthase was attributed to subunit dissociation induced by potassium iodide [39].

Interestingly, the same iodide quenching experiment on PLP-GSAM, carried out following the fluorescence emission intensity upon excitation at 420 nm, gives only small changes in the relative fluorescence intensity (data not shown). This suggests that the fraction of PLP present in the ketoenamine form is less accessible by the anionic quencher. This could be due in principle to a closed conformation of the active site or to a different arrangement of polar and charged residues at the active entrance with respect to the enolimine tautomer.

All together, the results obtained with PLP-GSAM indicate, in agreement with the crystallographic data, a degree of structural asymmetry in the microenvironment surrounding the enzyme-bound PLP. This asymmetry might correspond to an asymmetric distribution of the ketoenamine (less accessible) and enolimine forms of PLP at the two active sites of the enzyme.

**Figure 5:**
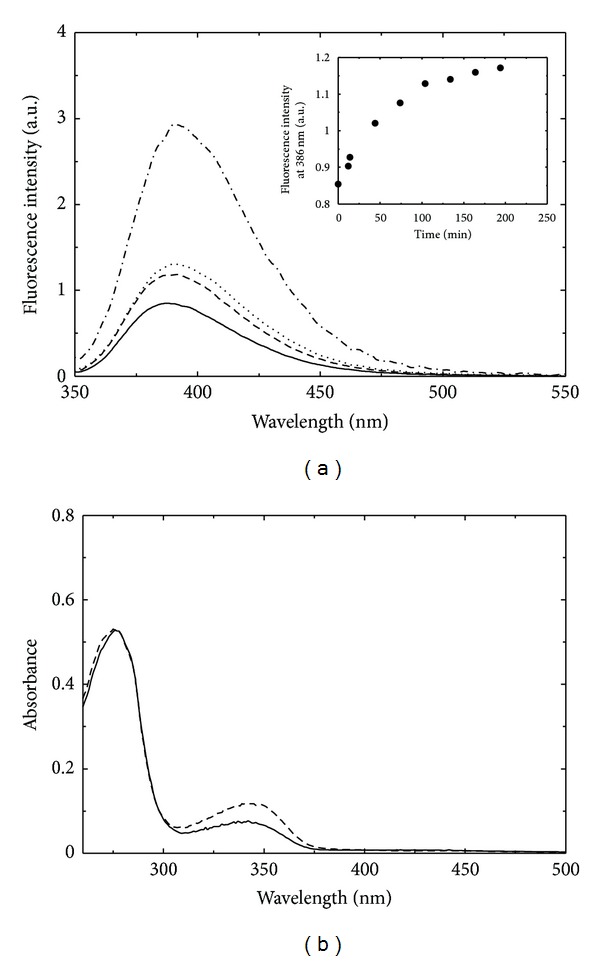
KI-induced release of the cofactor from PMP-GSAM. (a) Fluorescence emission spectra upon excitation at 330 nm of 2 *μ*M PMP-GSAM in the absence (solid line) and in the presence (dashed line) of 50 mM KI (after 195 minutes incubation). The emission spectra of 2 *μ*M PMP in Tricine (dashed-dotted line) and 1 *μ*M PMP in tricine plus 1 *μ*M PMP-GSAM (dotted line) are shown for comparison. Inset: time course of the fluorescence emission intensity of 2 *μ*M PMP-GSAM (*λ*_ex_ = 330 nm, *λ*_em_ = 386 nm) after the addition of 50 mM KI. (b) Absorbance spectrum of a solution of PMP-GSAM before (solid line) and after (dashed line) treatment with 50 mM KI. The protein solution was mixed with a concentrated solution of KI to a final concentration of 50 mM and extensively dialyzed first against 50 mM KI in tricine buffer pH 7.9 and then against KI-free buffer.

The dependence of the emission spectrum of PMP-GSAM on potassium iodide concentration is complex. The intensity of the emission upon excitation at 330 nm increases with increasing KI concentrations up to about 250 mM and then decreases with a red shift of the peak maximum (data not shown). Differently from what we observed on PLP-GSAM, the emission signal in the presence of KI is not stable with time, but, at low KI concentrations, it increases with time (Figure 5(a), inset). The fluorescence emission intensity of PMP free in solution is about 3 times higher than that of PMP bound to GSAM (Figure 5(a)). The increase of fluorescence emission caused by KI could thus be due to the release of the cofactor from the catalytic site, and the decrease and red-shifting of emission intensity at higher quencher concentration might be related to quenching of PMP free in solution. Indeed, the spectrum of 2 *μ*M PMP-GSAM after 195 minutes incubation in 50 mM KI nearly coincides with that calculated, adding the absorption of 1 *μ*M PMP in the presence of 50 mM KI to that of 1 *μ*M PMP-GSAM. This finding suggests that about 50% of the cofactor has been released from PMP-GSAM complex. This was confirmed by dialysis experiments in which a solution of PMP-GSAM containing 50 mM KI was first dialysed against 50 mM KI and then against KI-free buffer. After dialysis, the absorption peak of the cofactor was reduced by 1.6-fold (Figure 5(b)), further indicating that treatment with KI causes the release of about 50% PMP from PMP-GSAM. Other anions were previously shown to cause, at high concentration, the release of PMP from the active site, for example, GSAM from pea leaves treated with 0.2 M phosphate at acidic pH [43]. Interestingly, also in that case the release of PMP was not complete, about 44% of the theoretical amount.

Our results, showing that 50% of PMP dissociates from the enzyme at low KI concentration, suggest the release of PMP from only one active site and imply a structural asymmetry of the double PMP form of GSAM. This is in contrast with the previously published crystallographic data, showing that in the PMP-GSAM form, the active site loop is ordered and symmetrically opened in both subunits [15]. The asymmetry of PMP-GSAM in solution could be either preexisting or be generated once a single PMP molecule has left the dimer. The preexisting asymmetry could originate from the different experimental protocols used to prepare PMP-GSAM: by addition of PMP to the apoenzyme (crystallographic data), or by the addition of DAVA to the native enzyme, that is typically purified as an asymmetric PLP/PMP form (this work). In our PMP-GSAM, one active site of the dimer may still contain the reaction product. Indeed, we previously observed some instability, upon prolonged storage, of the PLP- and PMP-GSAM preparations obtained by reacting native GSAM with substrate analogues undergoing half-transamination reaction. Although from the absorption spectra, they appear to contain only one or the other form of the cofactor when freshly prepared, they seem to slowly revert to a form containing both PLP and PMP (data not shown). Interestingly, in the characterisation of different spectral forms of GSAM by electrospray mass spectrometry, Brody et al. [44] reported that reduction with NaBH_4_ of the PLP form yielded two molecular species of different mass, suggesting that the cofactor was actually present also as PMP. The PMP form obtained by the same authors was also recognised to have a “barely detectable” absorption peak at 420 nm.

## 4. Conclusion

The asymmetry of the active site loop conformation between subunits of dimeric glutamate-1-semialdehyde aminomutase in solution was investigated by fluorescence spectroscopy. Cationic and neutral quenchers are ineffective in quenching PMP-GSAM and PLP-GSAM fluorescence, likely due to the distribution of positive charges that characterizes the entrance of the active site of both enzyme forms. On the other hand, KI quenches PLP-GSAM fluorescence, although only the fraction of coenzyme molecules present as the enolimine tautomer is significantly accessible to the solvent, clearly indicating an asymmetric structure of the active sites. These observations match the crystallographic data, which show an asymmetric conformation of the loop controlling access to the active site, so that when the enzyme is in the double PLP form, one loop is closed and the other is disordered [15]. Due to the known effect of environment polarity on the equilibrium between the enolimine and ketoenamine forms of PLP, it is tempting to assume that selective quenching of the enolimine arises from asymmetric loop closure stabilizing a different equilibrium of tautomers at the two active sites. 

Comparatively low concentrations of KI cause the release of PMP from PMP-GSAM, apparently only from one subunit of the dimer. A possible interpretation of this result is that the double PMP-GSAM presents active site structural asymmetry, in contrast to previous crystallographic reports. Alternatively, this asymmetry may be generated once one PMP molecule has left the dimeric enzyme. This latter explanation is not in contrast with the crystallographic data and relies on the fact that communication between subunits of the GSAM dimer has been shown experimentally and has been proposed on the basis of structural and mechanistic considerations [15–17].

Other important examples of involvement of an active site gating loop in PLP-dependent catalysis are found in fold-type I decarboxylases, such as human isoforms of glutamate decarboxylase [45] and DOPA decarboxylase [46]. In these enzymes, an active site loop plays a pivotal role in controlling reaction specificity. In *O*-acetylserine sulfhydrylase, the interaction of the substrate carboxylate with a substrate-binding loop, highly conserved from bacteria to plants, triggers the closure of the active site to favor catalysis through proper positioning of substrate-binding groups and exclusion of water molecules [47–49]. Indeed, the control of substrate and reaction specificity, and catalytic efficiency, through an equilibrium between “open” and “closed” states of the active site appears to be a common feature in PLP-dependent enzymes [50].

## Figures and Tables

**Table 1:** Conformation of the active site loop in different crystal forms of dimeric *Synechococcus* GSAM [15].

Enzyme form	Loop at subunit 1	Loop at subunit 2
apo-GSAM	Disordered	Disordered
PMP-GSAM	Ordered, open	Ordered, open
PLP-GSAM	Ordered, closed	Disordered

## Abbreviations

ALA: 5-Aminolevulinate
DAVA: 4,5-Diaminovalerate
GSA: L-Glutamate-1-semialdehyde
GSAM: Glutamate-1-semialdehyde aminomutase
PLP: Pyridoxal 5*'*-phosphate
PLP-GSAM: GSAM form with PLP bound to both subunits of the dimer
PMP: Pyridoxamine 5*'*-phosphate
PMP-GSAM: GSAM form with PMP bound to both subunits of the dimer.
